# Photopic negative response of full-field electroretinography in patients with different stages of glaucomatous optic neuropathy

**DOI:** 10.1007/s10633-016-9528-z

**Published:** 2016-01-29

**Authors:** Marta Kirkiewicz, Wojciech Lubiński, Krzysztof Penkala

**Affiliations:** Clinic of Ophthalmology, Pomeranian Medical University, Powstańców Wlkp. Street 72, Szczecin, Poland; Department of Ophthalmology, Pomeranian Medical University, Szczecin, Poland

**Keywords:** Glaucoma, Photopic negative response, Scanning laser polarimetry

## Abstract

**Purpose:**

To evaluate photopic negative response (PhNR) discrimination ability between healthy and glaucomatous patients.

**Methods:**

Ninety eyes of 50 patients with primary open angle glaucoma (POAG) and 45 eyes of 23 healthy age- and sex-matched controls were investigated. Based on European Glaucoma Society criteria, POAG patients were divided into three groups: early, moderate and advanced glaucoma. Following measurements were analysed: mean defect (MD) from Humphrey Visual Field Analyzer, SITA standard 24-2 white on white perimetry; nerve fibre index (NFI) obtained from scanning laser polarimetry; and GDx and PhNR amplitude and PhNR/b-wave ratio. PhNR was elicited by red stimuli with flash strength of 1.6 cd s/m^2^ on blue background of 25 cd/m^2^. Correlations between retinal ganglion cells function (PhNR), retinal sensitivity (MD) and structure (NFI) were calculated. Sensitivity and specificity of PhNR parameters were calculated with standard formulas. Receiver operating characteristic (ROC) curves were used to determine optimal cut-off values. The area under the curve (AUC) was used to compare the ROC curves results between PhNR amplitude and ratio.

**Results:**

PhNR amplitude and ratio were significantly reduced in early, moderate and advanced glaucoma groups compared to controls. The sensitivity and specificity to detect glaucoma in early POAG were equal to 53.3 and 90.0 % for PhNR amplitude and 60.0 and 70.0 % for PhNR ratio; in moderate POAG 63.3 and 80.0 % for PhNR amplitude and 60.0 and 86.7 % for PhNR ratio; and in advanced POAG 76.6 and 80.0 % for PhNR amplitude, 90.0 and 73.3 % for PhNR ratio. There were no significant differences between AUC for PhNR amplitude (0.76–0.86) and PhNR ratio (0.78–0.86), *p* > 0.05. PhNR amplitudes and ratios correlated significantly with MD measured by SAP and NFI obtained from GDx (*p* < 0.05). PhNR amplitude significantly decreases with advancement of visual field defects in glaucoma patients.

**Conclusions:**

PhNR reveals dysfunction of RGCs in early, moderate and advanced stage of POAG. PhNR has good discrimination ability in detecting glaucomatous patients. PhNR might be a useful test in glaucoma diagnosis.

## Introduction

According to the World Health Organization, glaucoma is the second leading cause of preventable blindness globally [1]. Glaucoma diagnosis is still based on fundus examination, intraocular pressure (IOP) measurements and visual field testing. In many patients, visual field losses become detectable after a substantial number of RGCs has been lost [2, 3]. Previous studies have reported [3, 4] that repeatable defects in static visual field perimetry results occurred when at least 25–50 % of RGC had been lost. Damage of the RGCs is assessed with the use of imaging technologies. Optical coherent tomography (OCT) and GDx (scanning laser polarimetry) measure retinal nerve fibre layer (RNFL) thickness and can capture early morphological changes [5–7].

On the other hand, not only morphological changes, but also function of RGCs is important in glaucoma diagnosis and treatment. It is crucial to identify patients with early dysfunction of RGCs, before visual field loss and RGC damage occur.

It is possible to asses RGCs function with PhNR. PhNR is a negative-going wave that follows the b-wave of the photopic electroretinography. PhNR amplitude reflects averaged function of retinal ganglion cells (RGCs) population [8–10]. Viswanathan et al. [8] showed the reduction of this negative wave in mammals with experimental glaucoma (argon laser induced) and after tetradotoxin (TTX-sodium channels blocker) injection. Only few study results proved that PhNR amplitude in primary open angle glaucoma patients was reduced and this decrease in amplitude correlated with the degree of optic nerve damage represented by visual field loss [9–14]. However, Cursiefen et al. [15] suggested that PhNR could not distinguish so easy between glaucoma and healthy patients as it was previously showed on macaques.

That is why we decided to investigate discrimination ability of PhNR parameters in glaucomatous patients with different stages of POAG.

## Methods

### Subjects

Ninety eyes of 50 patients with POAG were enrolled in the study. They were recruited from ophthalmological outpatient clinics in Stettin, Poland. The diagnosis of POAG was based on glaucomatous disc morphology associated with visual field defects, measured by static automated perimetry (SAP, Humphrey Visual Field Analyzer, Model 750; Humphrey Instruments, San Leonardo, CA). The SITA standard strategy was applied to program 24-2 white on white (W–W)—mean defect (MD) was analysed. The visual field defect was described as glaucomatous based on European Glaucoma Society (EGS) guidelines [16] and classified into one of the three groups: early (MD > −6 dB), moderate (−12 dB < MD ≤ −6 dB) and advanced (MD < −12 dB) visual field defect. Three control groups for each glaucoma severity were selected from 45 eyes of 23 healthy controls. Both eyes were included in the study. Each control group consisted of 30 eyes sex-, refractive- and age-matched normal volunteers.

Optic nerve assessment was performed with scanning laser polarimetry with a version to variable corneal compensation (Gdx-VCC; Carl Zeiss Meditec, Inc., Dublin, CA). From GDx parameters, retinal nerve fibre indicator (NFI) was chosen to be analysed, because according to the literature it differentiates with the highest sensitivity and specificity between normal and glaucomatous patients [17, 18]. Patients’ characteristic is shown in Table 1.

**Table 1:** Groups characteristic: age, distance best corrected visual acuity, mean defect and nerve fibre indicator in patients with different stages of glaucomatous optic neuropathy and control groups

	*n*	Age (y)	DBCVA	MD (dB)	NFI
POAG 1	30	64.7 ± 9.7	0.03 ± 0.09	−1.6 ± 1.5	30.0 ± 13.9*
C	30	63.7 ± 9.1	0.03 ± 0.07	−1.0 ± 2.1	17.2 ± 6.4
POAG 2	30	67.0 ± 7.0	0.03 ± 0.09	−6.1 ± 2.5***	37.2 ± 18.9***
C	30	66.7 ± 7.8	0.05 ± 0.1	−1.2 ± 2.1	17.7 ± 7.7
POAG 3	30	66.8 ± 7.1	0.13 ± 0.15	−19.3 ± 6.8***	62.1 ± 24.3***
C	30	65.8 ± 7.3	0.06 ± 0.1	−1.1 ± 2.1	16.9 ± 7.8

Statistical significance: * *p* < 0.05; ** *p* < 0.01; *** *p* < 0.001; *POAG 1* early primary open angle glaucoma, *POAG 2* moderate primary open angle glaucoma, *POAG 3* advanced primary open angle glaucoma, *C* control group, *DBCVA* distance best corrected visual acuity, *MD*, mean defect, *NFI* nerve fibre indicator, data are presented as mean ± standard deviation

All patients gave and signed informed consent. The study was conducted according to the tenets of the Declaration of Helsinki and approved by the Local Ethical Committee.

### Electroretinography

**Fig. 1:**
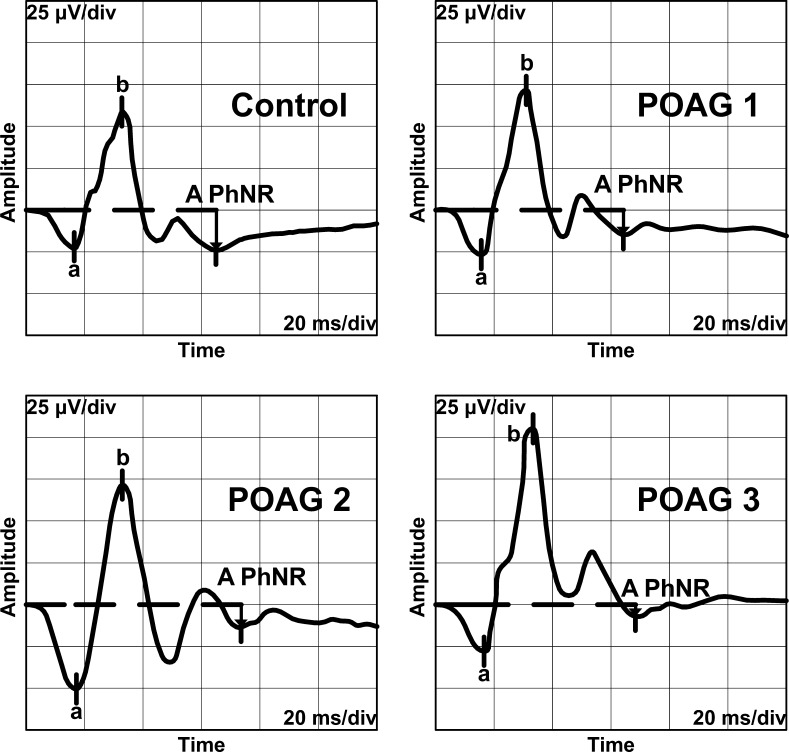
Representative traces of reduced PhNR amplitudes obtained from eyes with different stages of glaucomatous optic neuropathy in comparison with normal PhNR recording

PhNR was recorded binocular with corneal Dawson-Trick-Litzkow (DTL) electrodes. The reference electrodes were placed on a lateral canthi, and the ground electrode was attached to the centre of a forehead. Before the examination pupils were dilated by 10 % neosynephrine and 1 % tropicamide to minimum of 8 millimetres in diameter. Eyes were then adapted to the background light for 10 min. Stimulus conditions: a brief 4-ms red flash (640 nm, 400 cd/m^2^) at an intensity of 1.6 cd s/m^2^ against the blue background (450 nm) of 25 cd/m^2^ (photopic units). Signals were amplified and filtered with the band-pass filter of 1–300 Hz and recorded with full-field LED stimulator (RETI-port Roland Consult, 2003, Brandenburg, Germany). Forty responses were averaged, and the mean curve was analysed. The PhNR amplitude was measured from the baseline to the trough of negative peak following the b-wave (Fig. 1). PhNR/b-wave amplitude ratio was also calculated. This is a modified methodology of PhNR recording used by Viswanathan et al. [8, 9] and other authors [10, 13].

### Statistical analysis

Normality of data distribution was checked using the Shapiro–Wilk test. The results of two independent groups with normal distributed data were compared using Student’s *t* test. When at least one of the compared groups had not normal data distribution, the Mann–Whitney test was applied. Correlations of selected pairs were checked by calculating the Spearman’s correlation coefficient, because of not normal data distribution. To avoid inter-eye correlation, one eye from the same subject was randomly selected for analyse. Sensitivity and specificity of PhNR amplitude and ratio were calculated according to standard formulas. ROC curve was calculated in order to determine the cut-off point, for which the sensitivity and specificity of the test were the highest. The classification quality for PhNR was determine by measuring the area under the ROC curve—AUC. The level of significance of the test was set at 0.05.

## Results

Statistically significant reduction of PhNR amplitudes and ratios was observed in early, moderate and advanced glaucoma group. In Table 2 the mean PhNR amplitudes and ratios in POAG groups in comparison with control groups are shown. In Fig. 1 representative traces of PhNR in all presented stages of glaucomatous neuropathy are shown.

**Table 2:** PhNR mean amplitudes and ratios in examined groups of patients

	*n*	A PhNR [μV]	PhNR ratio
POAG 1	30	13.3 ± 9.2***	0.15 ± 0.1***
C	30	25.8 ± 15.7	0.35 ± 0.21
POAG 2	30	13.4 ± 11.8***	0.16 ± 0.13***
C	30	21.6 ± 11.2	0.31 ± 0.18
POAG 3	30	9.0 ± 6.1***	0.13 ± 0.13***
C	30	22.1 ± 11.0	0.32 ± 0.17

Statistical significance: *** *p* < 0.001; *POAG 1* early primary open angle glaucoma, *POAG 2* moderate primary open angle glaucoma, *POAG 3* advanced primary open angle glaucoma, *C* control group, *A* amplitude, *PhNR* photopic negative response, data are presented as mean ± standard deviation

When glaucomatous groups were compared between each other, no differences between means of PhNR amplitude and ratio in the early and moderate glaucoma groups (*p* > 0.05), as well as between moderate and advanced glaucoma groups (*p* > 0.05), were found. However, statistically significant difference was noticed between early and advanced group (*p* < 0.05).

**Fig. 2:**
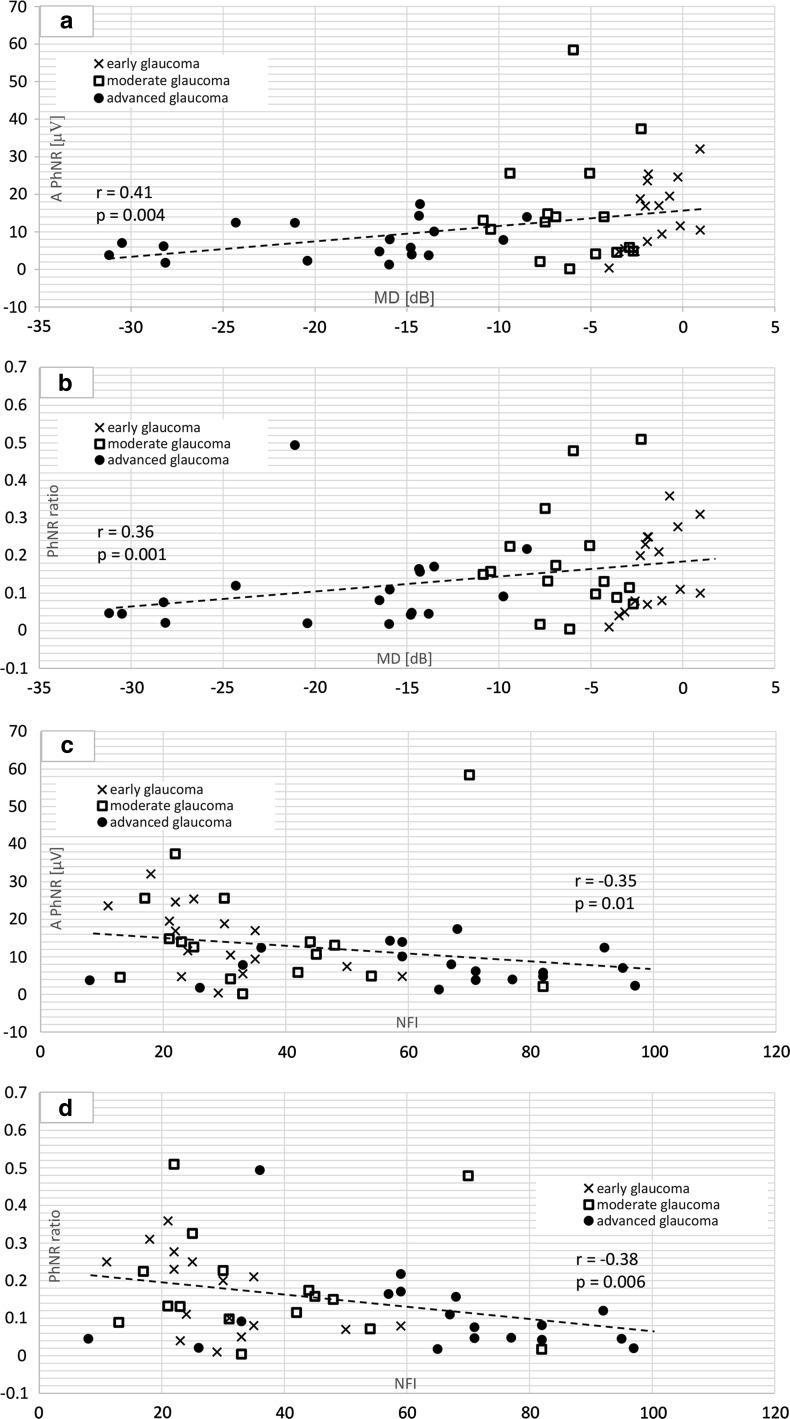
*Scatter plots* showing relationships between PhNR amplitude and mean defect (MD) (**a**); PhNR ratio and MD (**b**); PhNR amplitude and NFI (**c**) and between PhNR ratio and NFI (**d**) in glaucomatous group

Statistically significant correlation between the mean defect (SAP) and PhNR amplitude (*r* = 0.41, *p* = 0.004) and PhNR ratio (*r* = 0.36, *p* = 0.01) in glaucomatous patients was observed (Fig. 2). PhNR amplitude and ratio correlated significantly with NFI (*r* = −0.35, *p* = 0.01 for PhNR amplitude; *r* = −0.38, *p* = 0.006 for PhNR ratio). Correlations between structural parameter measured by GDx and PhNR are shown in Fig. 2.

**Fig. 3:**
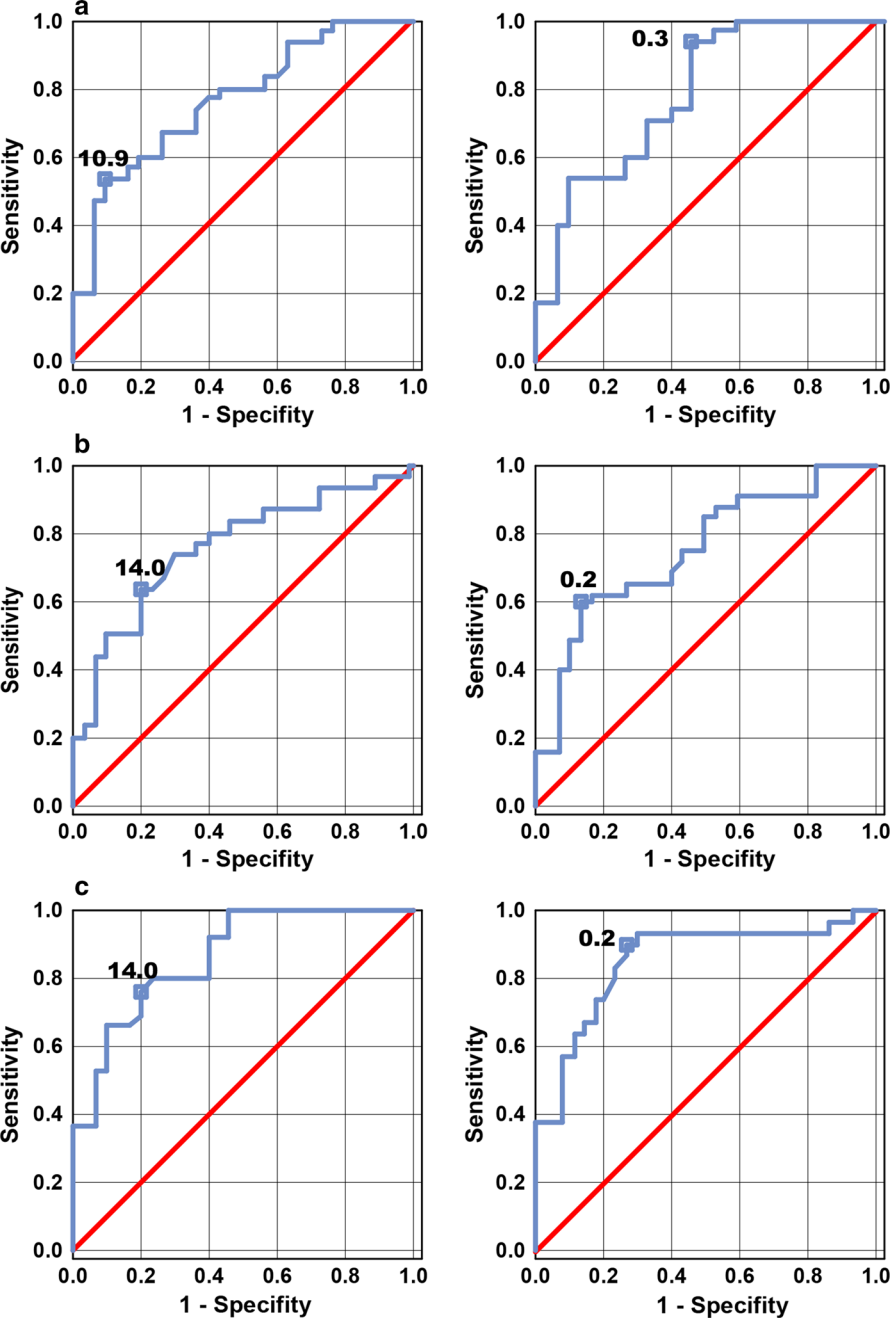
Receiver operating characteristic (ROC) curves for the PhNR amplitude (*left*) and PhNR ratio (*right*) in early (**a**), moderate (**b**) and advanced glaucoma (**c**)

Figure 3a illustrates ROC curves for PhNR amplitudes and ratios in early glaucoma group. Cut-off value for PhNR amplitude was equal to 10.9 μV and 0.3 for PhNR ratio. Using this point, the test had 53.3 % sensitivity and could estimate healthy from glaucoma patients with specificity equal to 90.0 %. For PhNR ratio, 70.0 % sensitivity and 60.0 % specificity were obtained.

In moderate glaucoma group cut-off values were equal to 14.0 μV for PhNR amplitude and 0.2 for PhNR ratio. PhNR sensitivity and specificity were equal to 63.3 and 80.0 % for amplitude and 60.0 and 86.7 % for ratio, respectively. AUC for amplitude and ratio was 0.8 (Fig. 3b).

In advanced glaucoma group cut-off values were again equal to 14.0 μV for PhNR amplitude and 0.2 for PhNR ratio. PhNR sensitivity and specificity were equal to 76.7 and 90.0 % for amplitude and 80.0 and 73.3 % for ratio, respectively. AUC for amplitude and ratio reached 0.9 (Fig. 3c).

There were no significant differences between AUCs for PhNR amplitude (0.76–0.86) and PhNR ratio (0.78–0.86) between groups of patients, *p* > 0.05.

## Discussion

The results of the presented study indicate that the PhNR recorded with the modified protocol of Viswanathan et al. [9] reveals the dysfunction of RGCs in patients with different stages of glaucomatous optic neuropathy. RGC function decreases gradually with severity of glaucomatous visual field loss.

The mean PhNR amplitude and ratio were reduced in early, moderate and advanced glaucomatous groups compared to healthy control. Even in patients with early glaucomatous optic neuropathy (POAG 1), PhNR reduction was significant and equal to 38 %. There are only two study results on PhNR in early glaucoma [14, 19]. North et al. [14] described 22 % of PhNR reduction compared to the control group with similar visual field loss measured by static automated perimetry (MD = −1.89 dB). Preiser et al. [19] found that even in patients with preperimetric glaucoma (average MD = 0.4 dB), reduction of the mean PhNR amplitude albeit not significant was noticed. Statistically significant (*p* = 0.0018) changes were observed only in glaucomatous group with greater mean sensitivity loss (average MD = −4.48 dB). There is only one research published by Machida et al. [20], who measured PhNR amplitudes in moderate (average MD = −8.8 dB) and advanced (average MD = −17.4 dB) optic neuropathy groups. They found 37 and 51 %, respectively, of mean PhNR amplitude reduction compared to the control group. These results are consistent with our data in the presented study. On the other hand, Cursiefen et al. [15] while examining patients with severe glaucomatous visual field loss (average MD = −13.0 dB) obtained a non-significant reduction of PhNR amplitude compared to the controls. The causes of these discrepancies are probable methodological differences: type of perimetry used for visual field testing (Octopus), different conditions of PhNR registration (white flash on a white background) and the type of used electrodes (Henkes).

PhNR test is able not only to graduate dysfunction of RGCs in different stages of glaucomatous optic neuropathy but also to demonstrate improvement of RGCs function after IOP reduction. Niyadurupola et al. [21] showed that PhNR amplitude improved in eyes of patients with different stages of glaucomatous optic neuropathy after IOP reduction of at least 25 %. In glaucoma patients, dysfunction of RGCs measured by PhNR was partially reversible even in advanced stage of the disease. The result of above-mentioned study suggests that PhNR might be a useful test in monitoring glaucomatous treatment.

Our results confirmed the occurrence of linear correlation between PhNR and the MD of SAP, which was previously described by Viswanathan et al. [9]. Other authors found that a curvilinear correlation model was a better fit than a linear regression [13, 22]. The fact of the correlation between PhNR and visual field defect in glaucomatous patients could be implemented in diagnosis process.

In this study we conducted the first comparative analysis of PhNR parameters and GDx (NFI). Statistically significant negative correlations between PhNR parameters and NFI were observed. There are few publications results showing relationships between PhNR and optic nerve structures measured by other methods like OCT [13, 22] and HRT [14]. The occurrence of these correlations is the additional indicator of the usefulness of PhNR in glaucoma diagnosis.

Table 3 summarises the results of PhNR and GDx parameters in different stages of glaucomatous optic disc neuropathy obtained from our study in comparison with data available in the literature. The sensitivity and specificity in detecting early glaucoma with PhNR (amplitude and ratio) ranged from 23.8 to 57.0 % and from 90.0 to 92.3 %, respectively [13, 20]. In moderate glaucoma, PhNR (amplitude and ratio) has better diagnostic ability and can distinguish glaucomatous eyes with sensitivity ranged from 40.7 to 88.0 %, whereas specificity reached 97.4 % [13, 20]. In advanced glaucoma, sensitivity of PhNR (amplitude and ratio) ranged from 66.7 to 93.0 % [13, 20], whereas specificity was equal to 92.3–97.4 % [20]. This comparison showed the compatibility of our results with previously published data.

**Table 3:** Comparison of PhNR and GDx sensitivities, specificities and AUCs in different stages of glaucomatous optic neuropathy

	POAG 1			POAG 2			POAG 3		
	A PhNR	PhNR ratio	GDx	A PhNR	PhNR ratio	GDx	A PhNR	PhNR ratio	GDx
Sensitivity (%)	38.1 [20]	23.8 [20]	23.0 [23]	59.3 [20]	40.7 [20]	80.0 [25]	66.7 [20]	69.7 [20]	90.1 [26]
	57.0 [13]	53.0 [13]	61.0 [17]	88.0 [13]	65.0 [13]	92.9 [26]	89.0 [13]	93.0 [13]	100.0 [25]
			76.0 [18]	83.0 [9]					
			82.0 [24]						
	**53.3**	**70.0**	**50.0**	**63.3**	**60.0**	**63.3**	**76.7**	**80.0**	**86.7**
Specificity (%)	92.3 [20]	97.4 [20]	62.0 [24]	92.3 [20]	97.4 [20]		92.3 [20]	97.4 [20]	
			91.0 [18]	90.0 [9]					
			95.0 [17]						
			96.0 [23]						
	**90.0**	**60.0**	**96.7**	**80.0**	**86.7**	**93.3**	**90.0**	**73.3**	**93.3**
MD ± SD (dB)	0.4 ± 0.2 [19]; −1.9 ± 0.4 [14] −3.2 ± 1.0 [12]; −3.3 ± 1.6 [20] POAG 1 [13, 17, 18, 23, 24]			−4.5 ± 5.7 [19]; −6.3 [9] −8.9 ± 1.6 [20]; −9.4 ± 2.8 [25] POAG 2 [13, 26]			−10.3 ± 6.3 [27]; −17.4 ± 4.5 [20]–20.5 ± 4.7 [25] POAG 3[13, 26]		
	−**1.6** ± **1.5**			−**6.1** ± **2.5**			−**19.3** ± **6.8**		
AUC	0.6 [19]	0.7 [20]	0.8 [24]	0.8 [19]	0.8 [20]	0.9–1.0 [26]	0.9 [20]	0.9 [20]	0.9–1.0 [26]
	0.7 [12, 20]	0.8 [19]	0.9 [17, 18]	0.9 [20]	0.8 [19]		1.0 [27]		
	0.8 [14]			1.0 [9]					
	**0.8**	**0.8**		**0.8**	**0.8**		**0.9**	**0.9**	

Mean defect of static perimetry is presented as mean ± standard deviation

*POAG* *1* early primary open angle glaucoma, *POAG 2* moderate primary open angle glaucoma, *POAG 3* advanced primary open angle glaucoma, *A* amplitude, *PhNR* photopic negative response, *GDx* glaucoma detection, *MD* mean defect, *SD* standard deviation, *AUC* area under the curve, bold letter: own data; square bracket numbers refer to the references list numbers

Nowadays measurement of RNFL by GDx, HRT and OCT is a valuable diagnostic method used in glaucoma. From the presented comparison of the data on PhNR and GDx in different stages of glaucomatous optic neuropathy, it is apparent that the PhNR may be equivalent to other diagnostic tests used in this disease.

In conclusion PhNR is a relatively new test in glaucoma diagnosis. It is worth mentioning that PhNR is obtained objectively as opposed to perimetry, which is heavily dependent on subject input. The results of the present study confirm results of previous published data indicating usefulness of PhNR in glaucoma diagnosis. However, it should be confirmed on a larger group of patients and a longer follow-up period. Recently in our opinion, this examination could be designed for glaucoma patients with low visual acuity, difficult to cooperate and should be reserved for diagnostically complicated cases.
